# Characterization of influenza virus variants induced by treatment with the endonuclease inhibitor baloxavir marboxil

**DOI:** 10.1038/s41598-018-27890-4

**Published:** 2018-06-25

**Authors:** Shinya Omoto, Valentina Speranzini, Takashi Hashimoto, Takeshi Noshi, Hiroto Yamaguchi, Makoto Kawai, Keiko Kawaguchi, Takeki Uehara, Takao Shishido, Akira Naito, Stephen Cusack

**Affiliations:** 1 Shionogi & Co., Ltd., Osaka, Japan; 2 European Molecular Biology Laboratory, Grenoble Outstation, Grenoble, France

## Abstract

Baloxavir acid (BXA), derived from the prodrug baloxavir marboxil (BXM), potently and selectively inhibits the cap-dependent endonuclease within the polymerase PA subunit of influenza A and B viruses. In clinical trials, single doses of BXM profoundly decrease viral titers as well as alleviating influenza symptoms. Here, we characterize the impact on BXA susceptibility and replicative capacity of variant viruses detected in the post-treatment monitoring of the clinical studies. We find that the PA I38T substitution is a major pathway for reduced susceptibility to BXA, with 30- to 50-fold and 7-fold EC_50_ changes in A and B viruses, respectively. The viruses harboring the I38T substitution show severely impaired replicative fitness in cells, and correspondingly reduced endonuclease activity *in vitro*. Co-crystal structures of wild-type and I38T influenza A and B endonucleases bound to BXA show that the mutation reduces van der Waals contacts with the inhibitor. A reduced affinity to the I38T mutant is supported by the lower stability of the BXA-bound endonuclease. These mechanistic insights provide markers for future surveillance of treated populations.

## Introduction

Influenza virus causes an infectious disease annually associated with 290,000 to 650,000 deaths and 3 to 5 million cases of severe illness worldwide^1^. In addition, pandemics, caused by newly emerging reassortment viruses, can have a devastating impact globally. Therefore, continued efforts are necessary to improve vaccines and anti-viral drugs as countermeasures^2^. Two classes of antivirals are currently available for clinical use, neuraminidase inhibitors (NAIs: oseltamivir, zanamivir, peramivir) and M2 ion-channel inhibitors (amantadine, rimantadine). However, circulating influenza viruses are now largely resistant to the M2 inhibitors^3^. Moreover, the antiviral potency of the NAIs is relatively moderate^4–7^, and another concern for this class of drugs is the emergence of resistance, as occurred during the 2008 to 2009 season when oseltamivir-resistant H1N1 was prevalent^8^. Therefore, more effective antiviral agents, with a novel mechanism of action, are required for the treatment and prevention of influenza virus infections.

The heterotrimeric RNA-dependent RNA polymerase (RdRp) of influenza virus is composed of subunits PA, PB1 and PB2. It is responsible for replication and transcription of the segmented, single-stranded viral RNA genome (vRNA) in the nucleus of infected cells^9,10^. Transcription of viral mRNAs occurs through a unique “cap-snatching” mechanism. This involves high-jacking host RNA polymerase II^11,12^ by binding of nascent capped transcripts to the PB2 subunit^13^ followed by cleavage at nucleotides 10–13 by the cap-dependent endonuclease (CEN) in the PA subunit^14,15^. The short, capped oligomers so generated serve as primers for transcription of viral mRNA by the RdRp function of the PB1 subunit^16,17^. The viral transcripts are poly-adenylated by a stuttering mechanism at a conserved U-rich region of the template vRNA for translation to functional proteins after nuclear export^18^. Since cap-snatching is essential for virus replication, the cap-binding^19^, endonuclease^20,21^ and RdRp activities are all attractive targets for small molecule inhibitors^22^, and indeed several novel compounds targeting the polymerase are under active clinical development^23^. Favipiravir (Avigan), an RNA synthesis inhibitor, was approved in Japan in 2014 although the indication is limited for treatment of novel influenza viruses unresponsive to other agents^24^. Pimodivir (JNJ-63623872, VX-787), a PB2 cap-binding inhibitor, has demonstrated virological efficacy alone and in combination with oseltamivir in uncomplicated influenza, but is only active against influenza A viruses^25,26^. Therefore, continued efforts to discover and develop influenza drugs with improved properties are still required.

**Figure 1:**
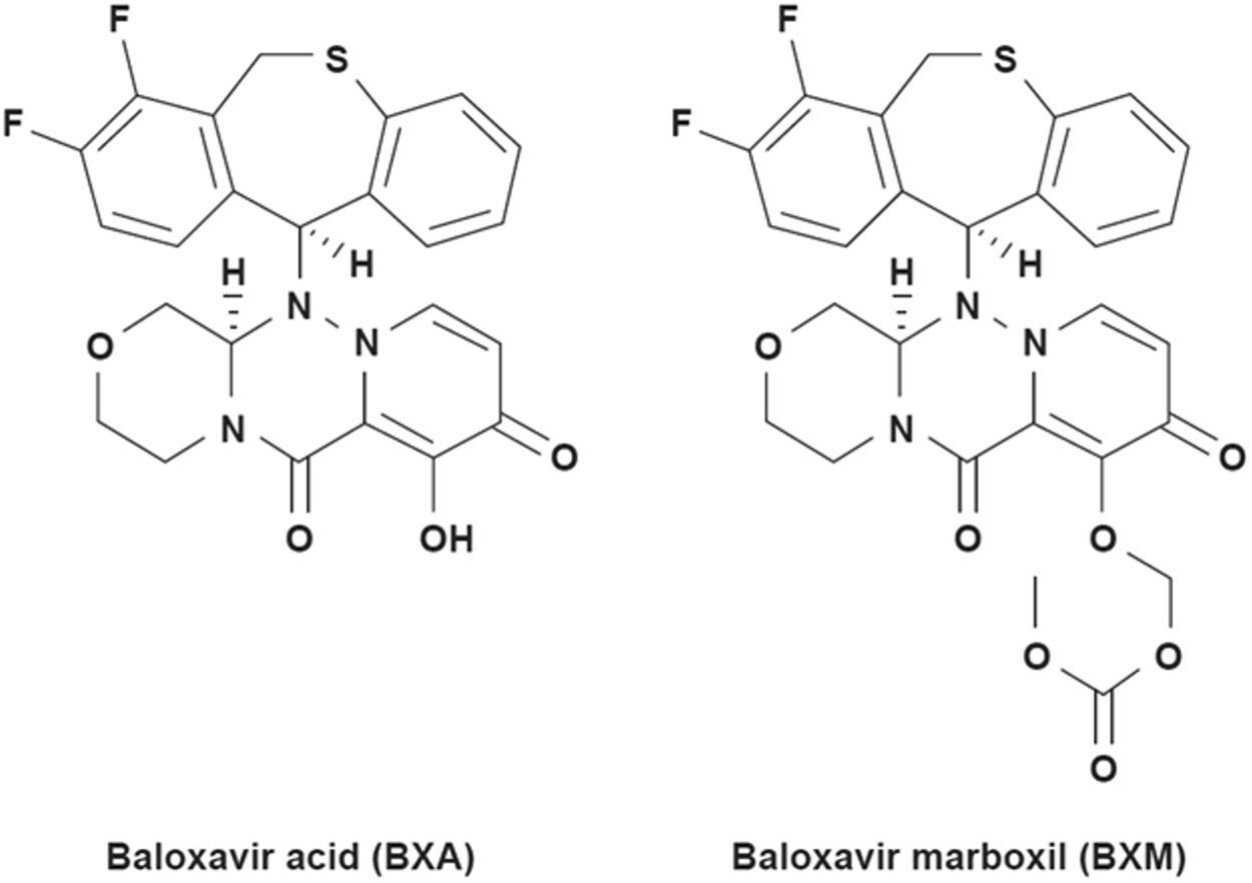
Chemical structure of baloxavir acid (BXA, active form) and baloxavir marboxil (prodrug form). The full chemical name of BXA is (12a*R*)-12-[(11 *S*)-7,8-difluoro-6,11-dihydrodibenzo[*b*,*e*] thiepin-11-yl]-7-hydroxy-3,4,12,12a-tetrahydro-1*H*-[1,4] oxazino[3,4-*c*]pyrido[2,1-*f*][1,2,4]triazine-6,8-dione.

As an alternative approach to influenza therapeutics, we developed baloxavir acid (S-033447; BXA) and its prodrug baloxavir marboxil (S-033188; BXM), in which a phenolic hydroxyl group was added to enhance oral absorption of BXA (Fig. 1). BXA was developed by rational molecular design based on the two-metal pharmacophore concept for dolutegravir (DTG), a strand transfer inhibitor of human immunodeficiency virus (HIV) integrase^27^. Both CEN and HIV integrase use two divalent metal ions as cofactors for their endonuclease activities. Since DTG binds to these ions in the HIV integrase active site, we proposed that the metal-chelating chemical scaffold of DTG could also be used for the development of CEN inhibitors. We initially screened metal-chelating compounds by CEN enzymatic assays, followed by a cellular phenotypic screen and then optimized the chemical structure to improve the pharmacokinetics and safety. This culminated in the generation of the compounds BXA and BXM that target the CEN in the PA proteins of influenza A and B viruses^23,28^. Because amino acids (AAs) in the active site of CEN are well conserved across seasonal, pandemic, and highly pathogenic avian influenza viruses^14,15^, BXA/BXM has the potential to be a new broad-spectrum, therapeutic class of anti-influenza therapy. Whereas single doses of BXM achieved a profound decline in viral titers of swabs, resulting in alleviation of influenza symptoms in uncomplicated influenza in clinical studies^23,28^, information on the mechanism of BXA binding to PA and potential determinants for reduced susceptibility are limited. Here, we report AA substitutions detected in the clinical studies of BXM and the impact they have on drug susceptibility and viral replicative capacity. To understand better the molecular mechanisms involved, we determined the co-crystal structures of wild-type and I38T endonuclease domains from influenza A and B viruses with bound BXA. Our virological and structural results highlight the importance of substitutions at PA residue Ile38 in the mechanism of reduced sensitivity to BXM and BXA.

## Results

### Resistance monitoring in phase 2 and pediatric studies of baloxavir marboxil

A multi-center, randomized, double-blind, controlled phase 2 study was conducted during the 2015-6 influenza seasons with BXM in Japanese adults aged 20–64 years with uncomplicated influenza (Japic CTI-153090). In the subsequent 2016-7 influenza seasons, an open-label study was conducted with BXM in otherwise healthy pediatric patients aged 6 months to <12 years with uncomplicated influenza (Japic CTI-163417). In the clinical trials, baseline variant monitoring was conducted to evaluate BXA susceptibility of the viruses in the baseline samples from nasopharyngeal/pharyngeal swabs. In addition, monitoring was performed in paired pre- and last positive swab samples from BXM treated patients to identify treatment-emergent AA substitutions that were associated with reduced susceptibility to BXA. In order to investigate the impact of the substitutions, reverse genetics was employed to generate the recombinant variant viruses in the corresponding influenza type/subtype, followed by drug sensitivity testing by means of plaque reduction with BXA and favipiravir or NA inhibition assays with oseltamivir acid (Table 1).

**Table 1:** Drug susceptibility of the recombinant viruses with amino acid substitutions to baloxavir acid, favipiravir and oseltamivir acid.

Strains	Baloxavir acid			Favipiravir			Oseltamivir acid		
	EC_50_ (nmol/L)		FC	EC_50_ (nmol/L)		FC	IC_50_ (nmol/L)		FC
	Mean	SD		Mean	SD		Mean	SD	
***Experiment 1***									
rgA/WSN/33 (H1N1)	0.42	0.12	N/A	17382.98	9432.24	N/A	0.80	0.09	N/A
rgA/WSN/33-NA/H274Y	0.32	0.06	0.77	11935.97	1201.47	0.69	181.65	5.13	**226.12**
rgA/WSN/33-PA/A36V	1.50	0.37	3.59	20707.85	14012.81	1.19	0.87	0.05	1.08
rgA/WSN/33-PA/V545T	0.31	0.11	0.73	12579.07	707.10	0.72	0.80	0.06	0.99
rgA/WSN/33-PA/I38T	11.37	1.85	**27.24**	7368.13	1531.62	0.42	0.80	0.01	1.00
rgA/WSN/33-PA/I38F	4.43	1.95	**10.61**	11254.26	3068.20	0.65	0.91	0.03	1.13
rgA/WSN/33-PA/A20S	0.50	0.27	1.19	12837.88	1190.52	0.74	0.83	0.10	1.04
rgA/WSN/33-PA/A20S + I38T	11.43	2.60	**27.38**	7995.78	1798.03	0.46	0.78	0.05	0.97
rgA/WSN/33-PA/A20S + I38F	3.38	1.16	8.10	9683.28	3488.79	0.56	0.78	0.09	0.97
rgA/WSN/33-PA/E23K	1.98	0.48	4.74	8941.88	2288.51	0.51	0.86	0.13	1.07
rgA/WSN/33-PA/E119D	2.70	1.50	6.46	9404.40	4090.35	0.54	0.85	0.09	1.05
rgA/Victoria/3/75 (H3N2)	1.13	0.51	N/A	8761.26	2968.09	N/A	0.13	0.02	N/A
rgA/Victoria/3/75-PA/A36V	6.87	2.76	6.09	9023.05	3510.61	1.03	0.16	0.01	1.16
rgA/Victoria/3/75-PA/I38T	63.80	3.40	**56.59**	5639.46	2773.00	0.64	0.16	0.01	1.21
rgA/Victoria/3/75-PA/I38F	22.69	10.82	**20.13**	9361.51	4923.51	1.07	0.14	0.01	1.06
rgA/Victoria/3/75-PA/E23K	6.20	2.86	5.50	5153.40	3832.48	0.59	0.17	0.01	1.27
rgA/Victoria/3/75-PA/E119D	5.09	2.48	4.51	4667.20	2395.22	0.53	0.14	0.01	1.02
rgB/Maryland/1/59	10.73	5.52	N/A	15696.09	7234.11	N/A	5.47	1.00	N/A
rgB/Maryland/1/59-PA/F36A	8.46	0.88	0.79	21918.27	15052.47	1.40	4.71	0.86	0.86
rgB/Maryland/1/59-PA/F36V	8.60	3.17	0.80	29606.21	12712.42	1.89	5.19	0.22	0.95
rgB/Maryland/1/59-PA/I38T	61.79	9.17	5.76	21815.70	13293.26	1.39	5.35	0.51	0.98
rgB/Maryland/1/59-PA/I38F	25.59	0.54	2.39	9611.08	3751.96	0.61	6.45	1.31	1.18
rgB/Maryland/1/59-PA/E23K	8.73	0.56	0.81	21503.05	13645.36	1.37	6.56	1.32	1.20
rgB/Maryland/1/59-PA/G548R	12.17	1.88	1.13	21505.25	9818.44	1.37	7.17	0.64	1.31
rgB/Maryland/1/59-PA/E120D	21.10	11.06	1.97	13337.46	1074.99	0.85	6.98	0.37	1.28
***Experiment 2***									
rgA/WSN/33 (H1N1)	0.31	0.11	N/A	12151.20	2034.53	N/A	0.93	0.10	N/A
rgA/WSN/33-NA/H274Y	N/T	N/T	N/T	N/T	N/T	N/T	194.13	36.42	**207.66**
rgA/WSN/33-PA/I38M	4.07	1.84	**13.15**	20150.53	14005.92	1.66	0.96	0.08	1.03
rgA/Victoria/3/75 (H3N2)	0.83	0.28	N/A	8625.24	3478.51	N/A	0.14	0.02	N/A
rgA/Victoria/3/75-PA/L28P	2.15	0.13	2.58	8454.41	4055.50	0.98	0.15	0.02	1.06
rgA/Victoria/3/75-PA/L28P + V63I	2.40	0.32	2.88	8885.88	1085.12	1.03	0.15	0.02	1.03
rgA/Victoria/3/75-PA/V63I	1.44	0.33	1.73	10268.05	2896.97	1.19	0.15	0.02	1.07
rgA/Victoria/3/75-PA/R356K	0.80	0.49	0.96	6977.99	1949.95	0.81	0.15	0.01	1.05
rgA/Victoria/3/75-PA/A37T	6.78	4.04	8.13	11323.06	1290.51	1.31	0.15	0.01	1.07
rgA/Victoria/3/75-PA/I38T	40.76	11.94	**48.90**	7868.86	810.03	0.91	N/T	N/T	N/T
rgA/Victoria/3/75-PA/I38T + E623K	35.34	16.12	**42.41**	3651.63	2226.60	0.42	0.14	0.01	1.00
rgA/Victoria/3/75-PA/I38M	11.48	1.43	**13.77**	12489.17	2100.63	1.45	0.14	0.01	1.03
rgA/Victoria/3/75-PA/N412D	0.45	0.02	0.54	5438.31	3166.98	0.63	0.13	0.01	0.93
rgA/Victoria/3/75-PA/V517A	0.43	0.22	0.52	4861.04	1205.27	0.56	0.15	0.01	1.03
rgA/Victoria/3/75-PA/E623K	1.00	0.29	1.20	7324.56	1807.50	0.85	0.14	0.01	0.98
rgA/Victoria/3/75-PA/S632P	0.61	0.28	0.74	5111.13	1956.91	0.59	0.15	0.01	1.05
rgA/Victoria/3/75-PA/E199G	3.72	1.37	4.46	8508.10	2292.85	0.99	0.13	0.01	0.89
rgB/Maryland/1/59	5.19	1.29	N/A	19481.73	4368.72	N/A	8.49	1.89	N/A
rgB/Maryland/1/59-PA/I38M	41.71	14.71	8.04	15519.49	3034.46	0.80	6.48	0.77	0.76
***Experiment 3***									
rgA/WSN/33 (H1N1)	0.45	0.22	N/A	14991.68	1395.47	N/A	1.03	0.25	N/A
rgA/WSN/33-PA/I38V	0.97	0.80	2.18	13027.98	2249.52	0.87	1.05	0.18	1.02
rgA/Victoria/3/75 (H3N2)	1.15	0.59	N/A	9391.13	695.68	N/A	0.12	0.01	N/A
rgA/Victoria/3/75-PA/I38V	2.11	0.81	1.83	11013.30	2541.61	1.17	0.15	0.01	1.23

EC_50_ of baloxavir acid and favipiravir were determined by plaque reduction assay, and IC_50_ of oseltamivir acid was evaluated by neuraminidase inhibition assay. Data represent the mean and standard deviation (SD) of more than three times of independent experiments. Fold change (FC) was calculated as relative EC_50_ of each tested virus to that of the cognate wild-type virus. Bold type, FC > 10; N/A, not applicable; N/T, not tested.

### BXA susceptibility of the viruses with amino acid changes detected in a phase 2 study

In the baseline monitoring of the phase 2 study, the viruses collected from nasopharyngeal/pharyngeal swabs were propagated in MDCK-SIAT1 cells to obtain sufficient quantity for phenotypic analysis^29^. The median EC_50_ of the baseline viruses to BXA ranged from 1.3 to 1.6 nmol/L for A/H1N1pdm, from 0.74 to 1.4 nmol/L for A/H3N2, and from 5.6 to 8.5 nmol/L for type B virus, in the BXM 10, 20 and 40 mg and placebo groups. In this setting, V36 and T545 in PA protein were detected in one patient each as polymorphic AAs from the viruses which showed more than 10-fold reduced sensitivity to the reference strain A/Victoria/361/2011(H3N2). Testing with recombinant rgA/WSN/33 (H1N1) viruses with A36V and V545T mutations showed that FCs in EC_50_ were 3.59 and 0.73-fold, respectively (Table 1). When A36V was introduced into rgA/Victoria/3/75 (H3N2), a 6.09-fold reduced susceptibility to BXA was observed, but no significant fold changes were observed when the AA at position 36 was changed in type B virus (Table 1). V36 was deposited at a frequency of 0.03% among H1N1 viruses in the NCBI database (Supp. Table 1), suggesting that polymorphic variants at position 36 are very rare, although they can slightly affect BXA susceptibility.

In the treatment-emergent variant monitoring of the phase 2 study, virus RNAs in the swab samples were extracted without virus amplification to avoid introduction of undesired mutations from 182 patients who had paired swabs available at baseline and the last positive in the BXM treated groups. The PA region was genotyped by the Sanger method at the lower limit of 1.03 × 10^5^ copy/mL for A/H1N1pdm virus, 1.52 × 10^5^ copy/mL for A/H3N2 virus, and 2.42 × 10^5^ copy/mL for type B virus. Treatment-emergent mutations in the PA region, which are defined as AA changes after a dose of BXM, were detected in five patients: I38T or I38F in two A/H1N1pdm-infected patients each and R548G in one type B-infected patient. In addition, PA genotyping was conducted on RNA extracted from post-treatment swabs from eight patients where an increasing viral titre compared to the previous time point was observed. This extensive sequencing resulted in the detection of the E23K change in PA of A/H1N1pdm virus for one patient. Susceptibility testing revealed that rgA/WSN/33 (H1N1) with the single substitution I38T, I38F or E23K showed FCs of 27.24, 10.61 and 4.74-fold, respectively, whereas the rgB/Maryland/1/59 with G548R mutation did not impact BXA sensitivity (Table 1). Since the viruses with I38F emerged during BXA treatment in one patient from a virus that originally possessed S20, the effects of A20S were assessed. Drug testing demonstrated that introduction of A20S into rgA/WSN/33 (H1N1) did not affect BXA sensitivity, and the FCs of combinations of A20S + I38T and A20S + I38F were comparable levels to single I38T or F mutations (Table 1). We also confirmed that A20S did not impact the replicative capacity of wild-type and I38F viruses (Supp. Fig. 1). Therefore, the polymorphic S20 did not affect reduced sensitivity to BXA caused by I38T or I38F changes.

Collectively, treatment-emergent monitoring in the phase 2 study identified I38T or I38F substitutions in A/H1N1 viruses as conferring more than 10-fold reductions in BXA susceptibility, whereas E23K had a significant but lesser impact. Given that I38 is highly conserved and that I38T and I38F were not detected in A/H1N1, A/H3N2 or type B viruses according to the NCBI database (Supp. Table 2), it is suggested that the A/H1N1 I38T or I38F viruses emerged as a result of exposure to BXM.

### BXA sensitivity of viruses with amino acid substitutions detected in a pediatric study

Similar monitoring was performed with the pediatric study in the subsequent influenza season. In the baseline monitoring, the viruses in nasopharyngeal/pharyngeal swabs were propagated in MDCK cells to obtain sufficient amount of virus stocks, and the EC_50_ values of BXA were determined by the ViroSpot assay^30^. The median EC_50_ values at baseline were 17.96 nmol/L for A/H1N1pdm, 4.48 nmol/L for A/H3N2, and 18.67 nmol/L for type B virus. In this setting, there was no sample which showed more than 10-fold reduction in BXA sensitivity compared to the reference strains. However, one sample from the A/H3N2 viruses-infected subjects showed a relatively low sensitivity to BXA with 7.75-fold reduction to the reference A/Victoria/361/2011(H3N2). Sanger sequencing identified specific PA polymorphisms P28 + I63 + R356, which were deposited in the NCBI database with the frequencies of 0.11%, 1.06%, and 99.32%, respectively (Supp. Table 1). Testing with BXA yielded FC values for L28P, V63I and L28P + V63I in A/H3N2 of 2.58, 1.73 and 2.88-fold, respectively, and mutation at position 356 did not have impact on BXA sensitivity (Table 1). Together, the AA substitutions that were identified by baseline monitoring in the pediatric study do not have a significant effect on BXA susceptibility.

In the treatment-emergent variant monitoring of the pediatric study, viral RNAs collected from 77 patients who had the paired swabs available at baseline and the last positive, were extracted without virus amplification to avoid introduction of unwanted mutations. The PA regions were genotyped by Sanger sequencing at the lower limit of sequencing defined of 4 log_10_10 (viral particle/mL). Treatment-emergent I38 variants were detected with total 15 A/H3N2-infected subjects, including twelve I38T and three I38M changes. Extensive sequencing of the viral RNA from 25 patients who exhibited rebound in viral titre resulted in identification of the I38T change in A/H3N2 virus for three patients. Drug testing showed that the FCs of A/H3N2 with the single mutation of I38T or I38M were 56.59 and 13.77-fold, respectively (Table 1).

E623K was detected along with I38T substitution in one patient. Therefore the impact of E623K and the combination of I38T + E623K on BXA susceptibility was evaluated with the conclusion that E623K had little impact. We also found that E623K did not affect the replicative capacity of wild-type and I38T viruses (Supp. Fig. 1). Apart from PA substitutions at position 38, A37T, E199G, N412D, V517A, and P632S were detected in one patient each. Testing demonstrated that A37T and E199G in A/H3N2 conferred slightly reduced susceptibility by 8.13 and 4.46-fold, respectively, whereas the mutations at positon 412, 517 and 632 did not impact on BXA susceptibility (Table 1).

Collectively, I38T and I38M in A/H3N2 viruses were identified by the treatment-emergent monitoring of the pediatric study as conferring more than 10-fold reductions in BXA susceptibility, whereas A37T and E199G had lesser effects. Since I38M is rarely found in the NCBI database, with frequencies of 0.02% and 0.04% in A/H1N1 and A/H3N2, respectively (Supp. Table 2), I38M presumably emerged because of exposure to BXM. We also confirmed that polymorphic I38V, which occurs at a frequency of 0.06% in A/H1N1, did not affect BXA sensitivity (Table 1 and Supp. Table 2).

### Effect of resistance mutations to other endonuclease inhibitors on susceptibility to BXA

It has been reported that PA E119D confers resistance to the compound L-742,001 that targets the CEN with a metal chelating mechanism similar to BXA^31^. We therefore evaluated the antiviral effect of BXA to type A and B viruses with the PA mutations E119D and E120D, respectively. We found that E119D conferred slightly reduced susceptibility to BXA by 6.46 and 4.51-fold in A/H1N1 and A/H3N2, respectively, while E120D in type B virus did not have a significant impact (Table 1). Of note, variants with E119D or E120D were not detected so far in the BXM clinical studies.

### Cross-resistance of BXA, favipiravir and oseltamivir acid

To test for cross-resistance, we evaluated the sensitivity to favipiravir with the panel of PA variant viruses. This revealed no cross-resistance (FC ranged from 0.42- to 1.89-fold, Table 1). Next, one of the NA inhibitor-resistant mutations, NA/H274Y, was introduced in A/H1N1 and the susceptibility to BXA was determined. Whilst NA/H274Y results in a more than 200-fold reduction in susceptibility to oseltamivir acid, BXA showed potent activity against these viruses. No significant cross resistance to the NA inhibitor was observed for any of PA variant viruses generated in this study (ranged from 0.76- to 1.31-fold, Table 1). Together, no cross-resistance of the viruses with less susceptibility to BXA was observed for favipiravir and oseltamivir acid.

### Replicative capacity of the viruses with amino acid substitution at I38 in PA protein

**Figure 2:**
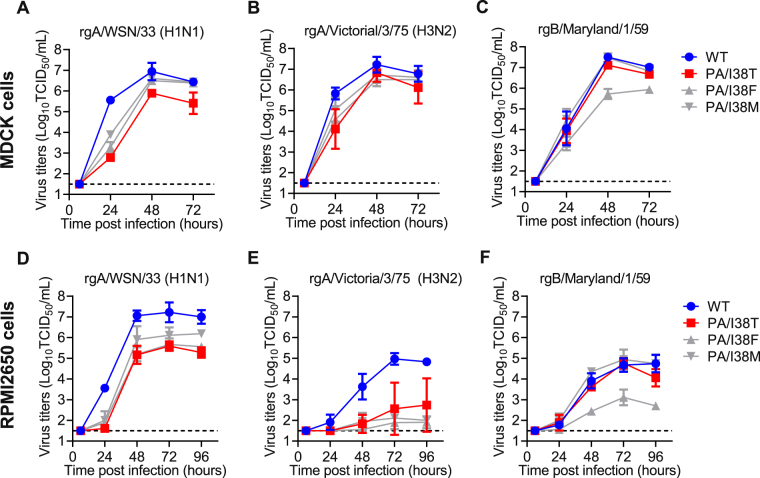
Replicative capacity of variant viruses with indicated AA substitutions in PA protein. Canine MDCK cells (**A**–**C**) or human RPMI2650 cells (**D**,**E**) were infected with WT or I38x viruses based on rgA/WSN/33 (H1N1) (**A**,**D**), rgA/Victoria/3/75 (H3N2) (**B**,**E**), or B/Maryland/1/59 (**C**,**F**). The culture supernatants were collected at the indicated time points and viral titers (TCID_50_/mL) were determined in MDCK cells. Each symbol represents the mean and standard deviation of triplicate experiments. The lower limit of quantification of the virus titers was indicated by a dashed line.

The results above suggest that substitution at I38 in PA protein is a major pathway for reduced susceptibility to BXA in the clinical setting, and I38T has the highest effect (30- to 50-fold). In order to investigate the impact of the I38T, I38F, I38M mutations on virus growth, the replicative capacity of the corresponding recombinant viruses was evaluated by measuring the time-course of viral titers in the supernatant of infected cell cultures. In MDCK cells, the viral titers of rgA/WSN/33 with I38T, I38F and I38M were lower than that of the wild-type (−2.78, −2.28 and −1.67 logTCID_50_/mL, respectively) at 24 hours post-infection, and similar results were obtained with rgA/Victoria/3/75 (H3N2) virus (Fig. 2A,B). In the case of rgB/Maryland/1/59, the viral titers of I38F were lower than that of the wild-type (−0.73 logTCID_50_/mL) at 24 hours post-infection, whereas those of I38T and I38M were comparable to the wild-type (Fig. 2C). Similar results were obtained when the human nasal squamous carcinoma cell line RPMI2650 was used (Fig. 2D–F). In summary, I38T, I38F and I38M substitutions in A/H1N1 and A/H3N2 had impaired replicative capacity compared to the wild-type in canine MDCK and human RPMI2650 cells. By comparison, for rgB/Maryland/1/59, I38F conferred impaired replicative capacity, whereas I38T and I38M viruses were comparable to the wild-type.

### *In vitro* endonuclease activity of wild-type and I38T endonuclease domain

**Figure 3:**
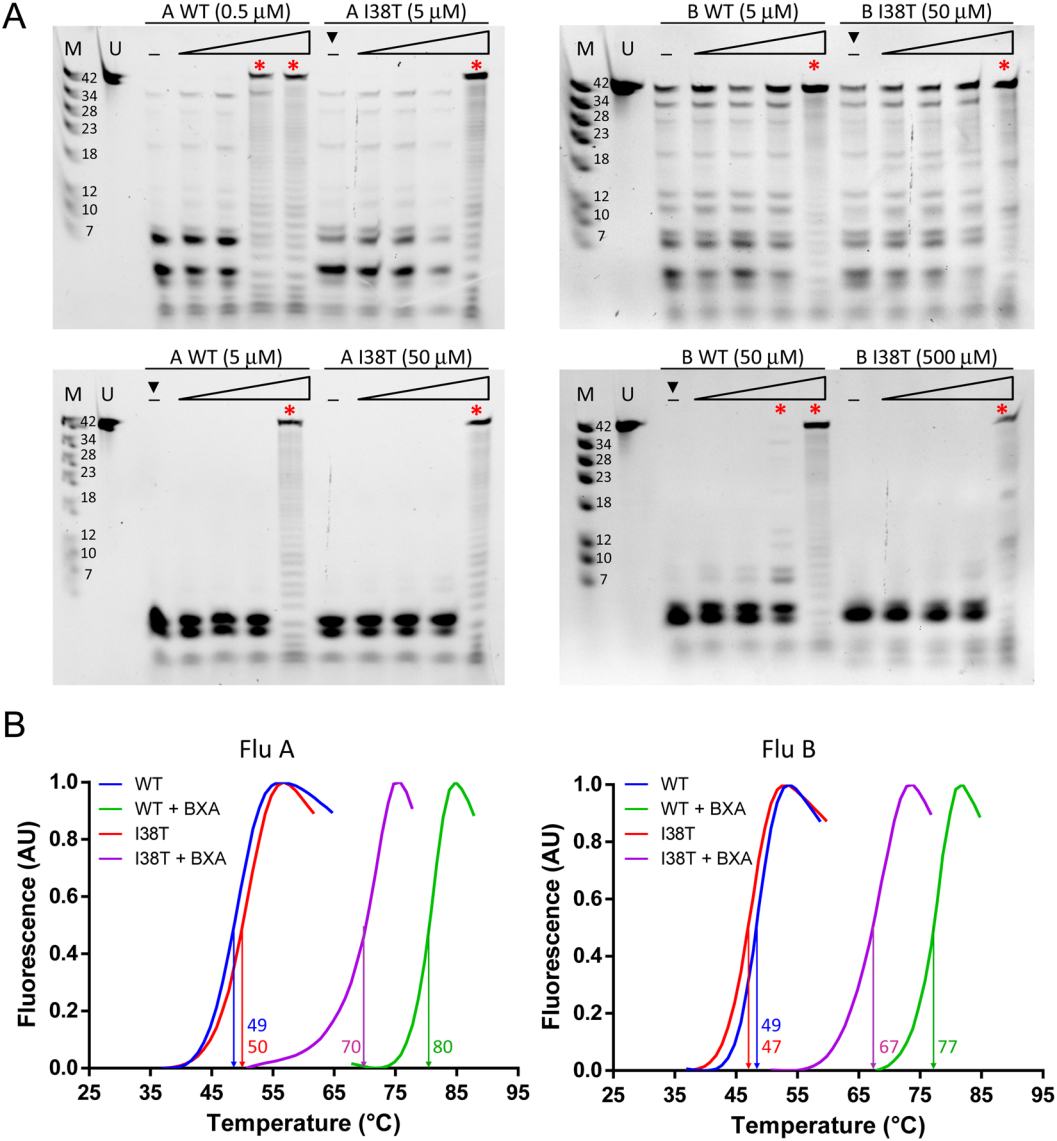
*In vitro* endonuclease activity and inhibition of PA variants and thermal stabilization induced by the binding of BXA. (**A**) For endonuclease activity (lane marked - in all panels), FAM-RNA was incubated with either 0.5–5 μM PA-A-WT, 5–50 μM PA-A-I38T, 5–50 µM PA-B-WT or 50–500 µM PA-B-I38T. Reactions were carried out for 2 hours in the presence of 2.5 mM MnCl_2_ at 37 °C and were stopped by adding 1 mM EDTA. For inhibition assays, all four proteins were pre-incubated with increasing concentrations of BXA for 30′ at RT and then tested for activity. In all cases, four compound concentrations were tested, maintaining the molar ratio to protein equal to 0.1–0.2–0.5–1 (in all panels, WT: lanes 4–7, I38T: lanes 9-12). The reaction products were loaded on an 8 M 20% acrylamide gel and detected at 520 nm using standard emission filter in Gel Doc™ XR+ (BioRad). Images were processed using Image Lab software (v 6.0.0.25, BioRad) as detailed in Methods. Gels were cropped (removal of gel wells and band-less edges) for clarity. Black triangles (▼) compare the RNase activity of WT and I38T forms at equal protein concentration for FluA (5 µM, left) and FluB (50 µM right). Red asterisks (*) indicate the BXA concentration at which RNase activity is significantly reduced or abolished. For all panels, lane 1 “M”: molecular ladder (FAM-RNAs; numbers represent the RNAs size in nt). Lane 2 “U”: uncleaved RNA, input substrate. (**B**) BXA binding stabilizes PA WT (blue, green) and PA I38T (red, purple) for both influenza A (left) and influenza B (right). Thermal shift assays were performed with 13 µM PA (purified with buffer supplemented with 2 mM MnCl_2_ and 2 mM MgCl_2_) in 20 mM Tris/HCl pH 7.5, 100 mM NaCl in the presence or absence of 0-1.3 mM of BXA and SYPRO Orange dye (Invitrogen). The curves represent the fluorescence emission of the dye at 575 nm during protein denaturation. Green and purple curves represent the stabilization effect induced by BXA at concentration ≥36 μM in complex with WT and I38T constructs, respectively. The estimated Tm values, taken as mid-points between local minima and maxima, are indicated and listed in Table 2.

To verify the reduced endonuclease activity of the I38T mutant, we purified the isolated endonuclease domain from influenza A and B virus polymerase with either Ile or Thr at position 38. For influenza A, we used residues 1–198 from strain A/California/04/2009 (pH1N1) with truncation of the flexible loop (Δ52–64:Gly), as previously described^21^. For influenza B, we used residues 1–197 from strain B/Memphis/13/03, as previously described^32^. Supp. Fig. 2 shows that the sequences of these strains differ at a few positions from those used in the recombinant virus studies but these natural variant amino acids are not close to the endonuclease active site and are not expected to influence the mode of BXA binding. Using a FAM-labelled 42 nt ssRNA substrate we first compared the nuclease activity of FluA wild-type and I38T endonuclease domains under identical conditions. A similar experiment was done for FluB but using 10 times higher protein concentration due to the significantly lower endonuclease activity of FluB compared to FluA^32^. The results show a 10-fold reduced activity of the I38T mutant endonuclease *in vitro* for both FluA and FluB (Fig. 3A, black triangles). We then tested the effect of BXA binding on the endonuclease activity by incubating WT or I38T PA from FluA or FluB with increasing concentrations of inhibitor. To take account of the reduced activity of the mutant, for each strain we conducted two sets of experiments in which protein concentrations of WT or I38T were adjusted to allow an identical starting RNA degradation activity. The inhibitor-to-protein ratios were identical in all tests. For both FluA and FluB, the endonuclease activity of the I38T mutant was inhibited only at the highest BXA concentration (1:1 ratio), whereas WT forms showed reduced or no activity already at 0.5:1 BXA:protein ratio (Fig. 3A, red asterisks). Taken together, these results are in line with the observed reduced replicative capacity and reduced sensitivity to BXA of mutant viruses bearing the AA change I38T (Table 1), with a more marked effect on FluA than on FluB. However this analysis does not allow quantitative derivation of IC_50_ or Ki.

### Thermostabilisation of the wild-type and I38T endonuclease upon BXA binding

The effect of BXA binding on the thermal stability of wild-type and I38T endonuclease domains from both FluA and FluB was measured in solution using the Thermofluor assay^33^. Melting curves and the associated melting temperature (Tm) of the proteins both in absence and in presence of BXA were measured (Fig. 3B, Table 2). In the absence of BXA, the Tm of all four endonuclease constructs is 46–50 °C. BXA binding to the endonuclease increases very significantly its thermal stability, with the Tm for the wild-type FluA or FluB endonuclease shifting to 78 or 77 °C, respectively, corresponding to ΔTm of +32 and +29 °C, respectively. Similar effects have been reported before for other endonuclease inhibitors but not of such high magnitude^14,21^. The large shifts imply BXA binds to FluA or FluB endonuclease with high affinity forming a stable protein-inhibitor complex. In contrast, the Tm of the T38 mutants was significantly lower, being 70 and 67 °C for Flu A and FluB respectively (ΔTm of +20 °C for both). The ~10 °C less thermal stabilisation of the mutant compared to the wild-type endonuclease is consistent with BXA being less tightly bound by the I38T variant endonucleases.

**Table 2:** Thermal stabilization of influenza A and B, wild-type and I38T mutant, endonuclease domains by BXA. For all samples, n ≥ 3.

	0 μM^a^	≥36 μM	ΔTm
PA-A WT	46 ± 3^b^	78 ± 2	+32
PA-A I38T	50 ± 0	70 ± 1	+20
PA-B WT	48 ± 1	77 ± 0	+29
PA-B I38T	47 ± 0	67 ± 1	+20

^a^Concentrations refer to BXA.

^b^Concentrations refer to BXA.

### Structural analysis of influenza A and B WT and I38T endonuclease with bound BXA

To give structural insight into the mechanism of reduced susceptibility of the I38T variant, we co-crystallised BXA with the PA endonuclease domain from influenza A and B virus polymerase with either I38 or T38. The four co-crystal structures were determined at 1.8 to 2.3 Å resolution (Table 3). In addition, a structure of the influenza B domain with the I38T mutation was determined without bound compound at 1.8 Å resolution (Table 3). A structure of the wild-type I38 influenza A domain without ligand was published previously^21^ (PDB:4AVQ).

**Table 3:** Crystallographic data collection and refinement statistics.

	A/H1N1 WT I38 + BXA	A/H1N1 I38T + BXA	B/Memphis WT I38 + BXA	B/MemphisI38T + BXA	B/Memphis I38T
PDB ID	6FS6	6FS7	6FS8	6FS9	6FSB
**Data collection** ^**a,b**^					
Data collection date	23/11/17	18/10/17	25/09/17	18/10/17	04/10/17
Beamline	ID30A-3	ID30A-1	ID29	ID30A-1	ID30A-1
Wavelength (Å)	0.968	0.966	1.254	0.966	0.966
Space group	*P* 2_1_ 2_1_ 2	*P* 2_1_ 2_1_ 2	*P*4_3_	*I*4_1_	*P*2_1_
**Cell dimensions**					
*a*, *b*, *c* (Å)	135.2, 75.7, 121.6	135.7, 75.6, 121.8	59.8, 59.8, 125.9	63.7, 63.7, 125.4	38.0, 61.5, 92.6
α, β, γ (°)	90, 90, 90	90, 90, 90	90, 90, 90	90.0, 90.0, 90.0	90, 95.5, 90
Molecules per ASU	6	6	2	1	2
Resolution range (Å)	90.41-2.29	90.65-1.96	59.86-1.80	56.81-2.28	50.0-1.80
*R*_*merge*_	0.083 (0.908)	0.043 (0.596)	0.064 (0.471)	0.044 (0.561)	0.055 (0.580)
*I*/σ*I*	14.8 (2.2)	17.4 (2.2)	13.6 (2.6)	16.1 (2.4)	7.55 (1.2)
Completeness (%)	99.9 (100)	99.4 (99.7)	91.8 (93.7)	93.5 (99.5)	91.3 (92.4)
Redundancy	6.9 (7.2)	4.1 (4.0)	3.6 (3.4)	4.4 (4.4)	1.7 (1.7)
CC1/2	0.999 (0.770)	0.999 (0.767)	0.998 (0.862)	0.999 (0.899)	0.996 (0.596)
**Refinement** ^**b**^					
Resolution (Å)	2.29	1.96	2.00	2.30	1.80
No. reflections work/free	56754/2796	89882/4372	35748/1758	10147/516	37473/1863
*R*_work_	0.193 (0.256)	0.188 (0.255)	0.181 (0.260)	0.179 (0.272)	0.197 (0.327)
*R*_free_	0.231 (0.320)	0.211 (0.321)	0.209 (0.300)	0.235 (0.372)	0.236 (0.351)
No. atoms (total)	9209	9706	3277	1540	3457
Protein	8796	8987	3019	1494	3329
Ligands	204	204	68	34	—
Solvent	197	503	186	10	125
Divalent cations	12	12	4	2	3
Average *B*-factor (Å^2^)	51.9	42.5	24.7	64.1	36.2
**R.m.s. deviations**					
Bond lengths (Å)	0.004	0.004	0.010	0.016	0.011
Bond angles (°)	0.847	0.799	1.436	1.824	1.043
**Ramachandran (%)**					
Favoured	97.8	97.6	98.9	96.1	99.8
Outliers	0.20	0.0	0.28	0.56	0.0
**Molprobity**					
Clash score	1.47	1.89	1.80	3.98	1.82
Molprobity score	1.02	1.26	1.22	2.01	1.24

^a^For each structure, one crystal was used.

^b^Values in parentheses are for the highest-resolution shell.

**Figure 4:**
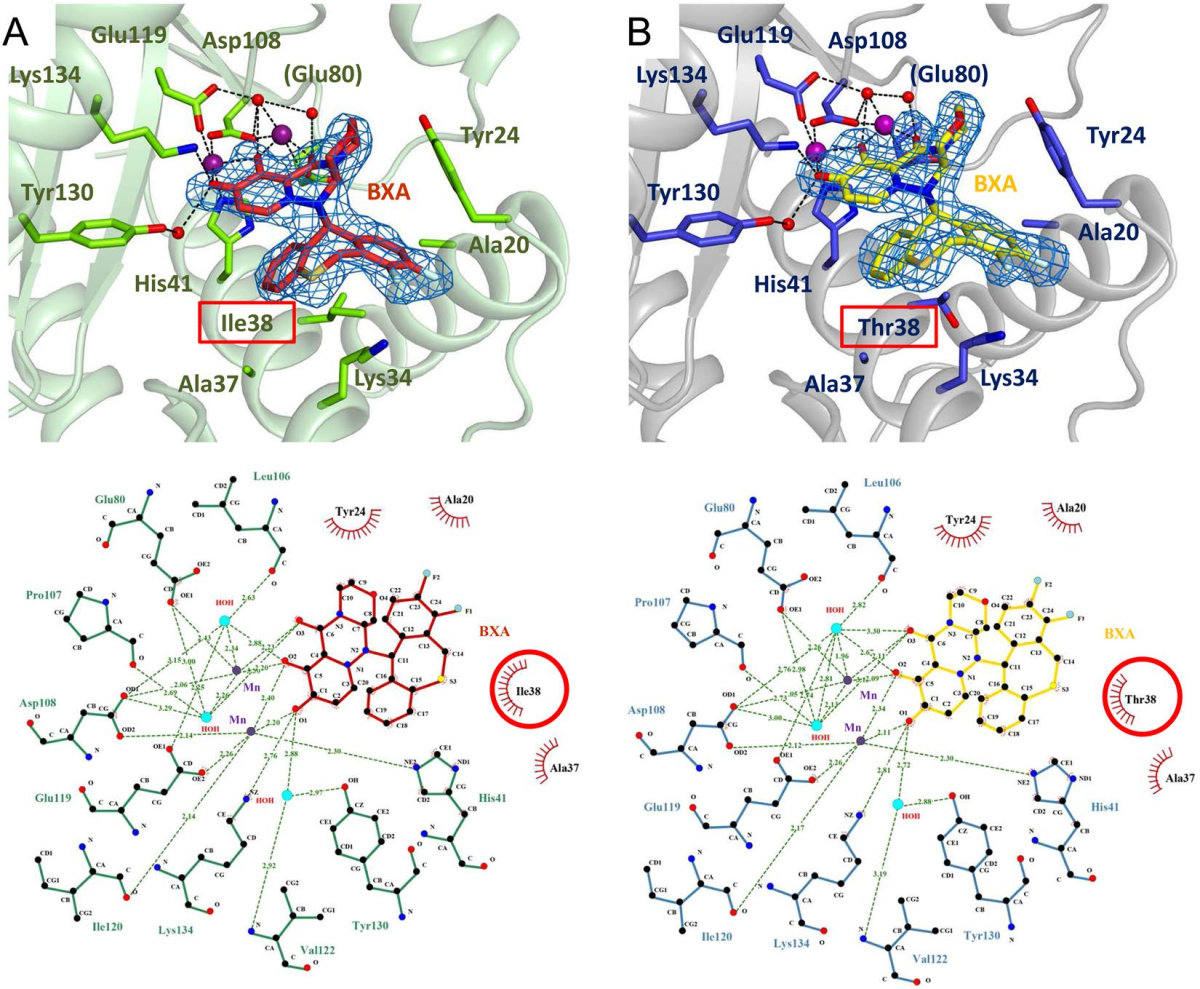
BXA binding to influenza A/H1N1 PA endonuclease. BXA interacts with (**A**) PA-A WT and (**B**) PA-A I38T by chelating the two manganese ions in the active site. Top panels represent the 3D structure of BXA-bound PA-A endonuclease, whereas the bottom ones are the corresponding 2D interaction ligplots. In the structures, both BXA (in TV red for PA WT and yellow for PA I38T) and the interacting side chains (in chartreuse green for WT and TV blue for I38T) are represented as sticks, Mn ions (purple) and water molecules (red) as spheres. In blue mesh, the omit Fo-Fc density map for BXA at 3.0 σ. In the ligplots, dash lines represent hydrogen bonds, red crowns hydrophobic interactions and red circles highlight the mutation site at position 38. Structural figures were prepared using Pymol (www.pymol.org), ligand interaction plots with LigPlot+ (EMBL−EBI).

**Figure 5:**
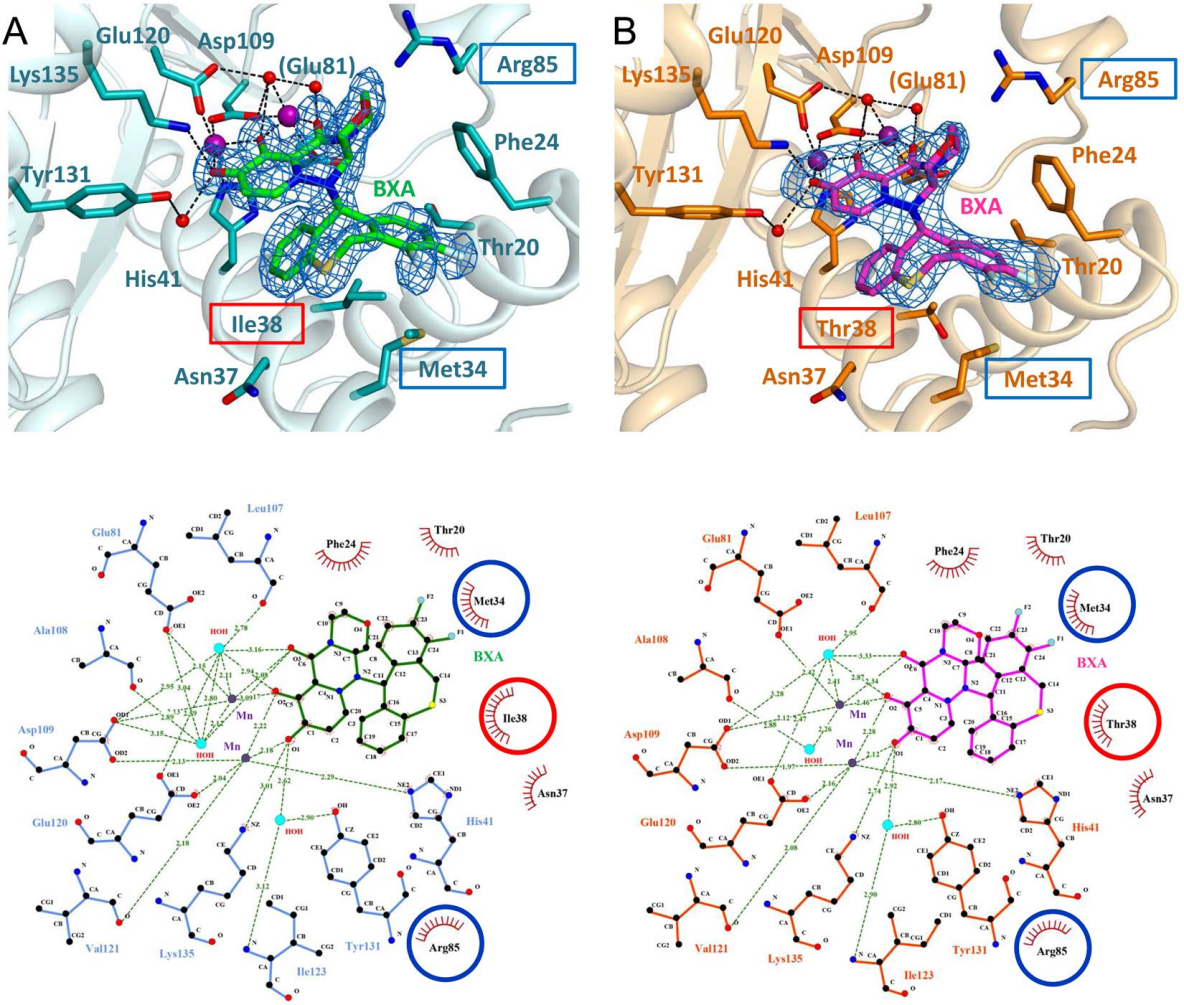
BXA binding to influenza B/Memphis PA endonuclease As Fig. 4, but for (**A**) PA-B WT and (**B**) PA-B I38T. Top panels: 3D structures of BXA-bound PA-B. Bottom panels: corresponding 2D interaction ligplots. Both BXA (in green for PA WT and light magenta for PA I38T) and the interacting side chains (in teal for WT and orange for I38T) are represented as sticks, Mn ions (purple) and water molecules (red) as spheres. The protein main chain is drawn as a cartoon. Represented by the blue mesh is the omit Fo-Fc density map for BXA at 3.0 σ. In the ligplots, dash lines represent hydrogen bonds and red crowns hydrophobic interactions. Red circles highlight the mutation site, blue circles the additional hydrophobic contacts that characterize binding of BXA to PA-B. Ligand interaction plots were prepared with LigPlot+ (EMBL-EBI).

**Figure 6:**
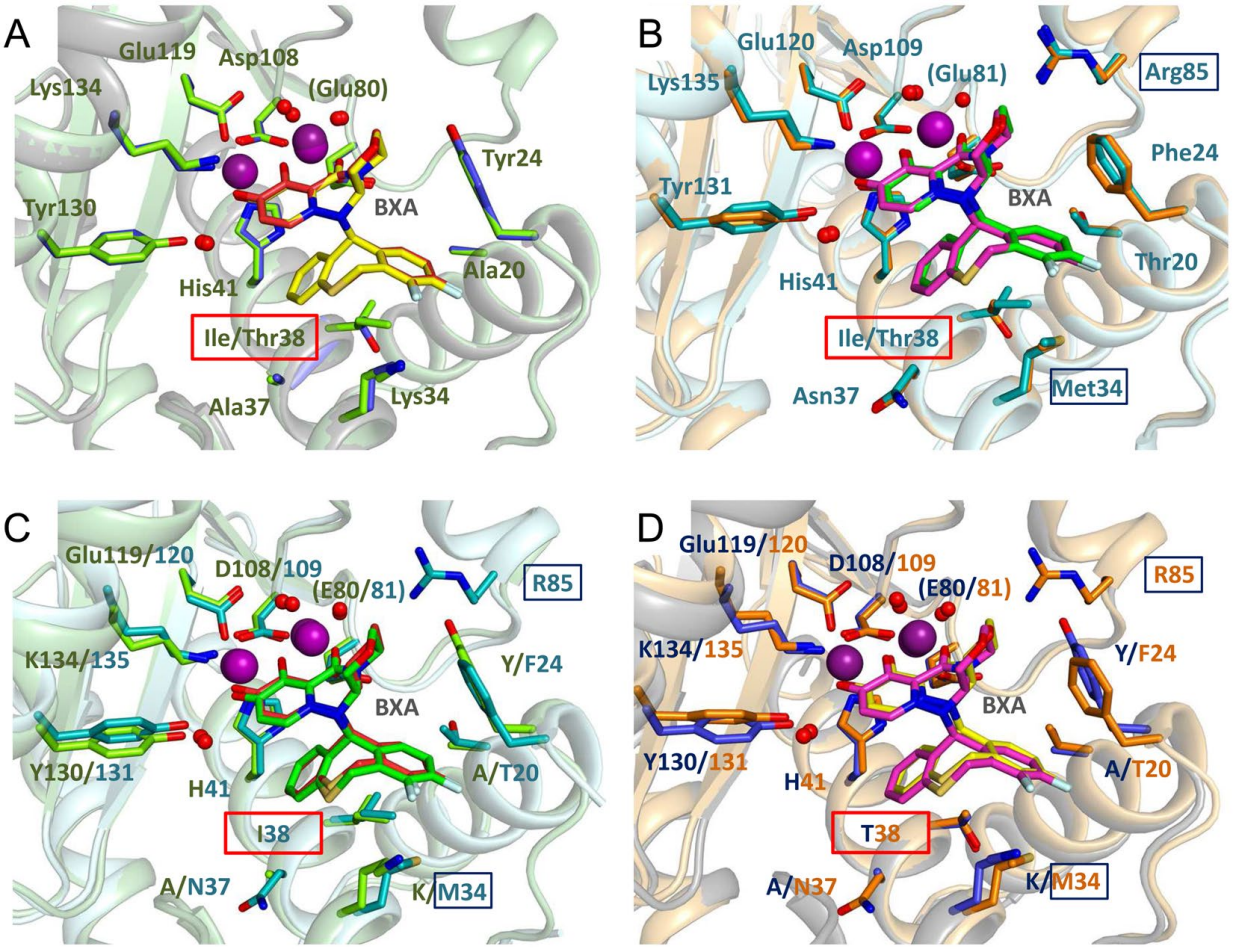
Comparison of PA endonuclease from Flu A and Flu B bound to BXA in either WT or I38T form. Superposition of PA-BXA complexes: (**A**) PA-A WT and PA-A I38T, (**B**) PA-B WT and PA-B I38T, (**C**) PA-A WT and PA-B WT, (**D**) PA-A I38T and PA-B I38T. Complexes PA/BXA are represented in sticks and coloured in chartreuse/TV red for A WT, TV blue/yellow for A I38T, teal/green for B WT, orange/light magenta for B I38T. Manganese ions and water residues involved in protein-inhibitor interactions are depicted as spheres, in purple and red, respectively. Protein main chain is represented as a cartoon. Highlighted in red rectangles are the mutation sites. The binding of the inhibitor does not differ significantly among the four protein constructs, which share high conservation in the local network of interactions with BXA, except locally when residue 38 is mutated. Additional contacts in the case of Influenza B are shown in blue rectangles in B, C and D.

All four PA-BXA co-crystal structures exhibit clear and unambiguous electron density for the compound, which is bound in essentially the identical fashion in each structure (Figs 4–6). BXA has a butterfly like form (Fig. 1), with one wing comprising a metal chelating polar oxazino-pyridotriazin-dione head-group and the other wing, a lipophilic, difluoro-dihydro-dibenzothiepine tail-group that makes van der Waals contacts with certain residues of the active site pocket. As expected, the head-group binds to the two divalent cations in the endonuclease active center, each metal ion having octahedral co-ordination through interactions with the protein, to the compound or water molecules, as previously described^21^. The crystals were grown in the presence of 2 mM Mg^2+^ together with 2 mM Mn^2+^, with only the latter having detectable anomalous scattering at the wavelength used. Whereas anomalous difference maps show peaks at both metal sites, that at site 1 (co-ordinated by His41, Glu119 and Asp108 in PA-A) is consistently about twice the height of that at site 2 (co-ordinated by Asp108 and Glu80 in PA-A) (Supp. Fig. 3). Moreover, when site 2 is refined as a manganese, the B-factor is consistently higher than the co-ordinating atoms. This is consistent with previous observations that site 1 preferentially binds manganese whereas site 2 is partially occupied by both manganese and magnesium^21,34^.

**Figure 7:**
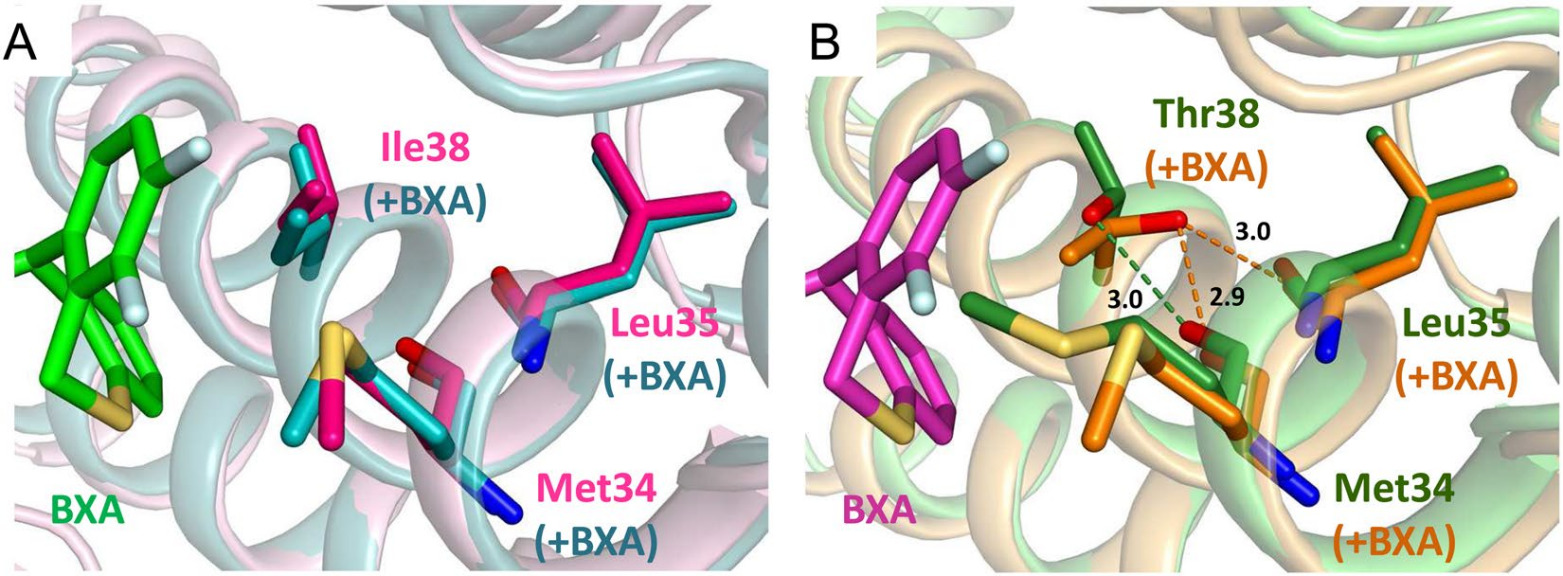
Local interactions of residue 38 in apo- and BXA-bound FluB PA (**A**) Superposition of ligand-free PA-B WT (PDB:5FML, in hotpink) and bound to BXA (green sticks for BXA, teal sticks/cartoon for PA). (**B**) Superposition of ligand-free (forest green) and BXA-bound PA-B I38T (light magenta sticks for BXA, orange sticks/cartoon for PA). For the WT, BXA binding does not alter the disposition of Ile38 and neighbouring Met34. For the I38T mutant, both side-chains change rotamer and the contacts with both the main chain and the compound hydrophobic pocket are altered.

The V-shaped tail-group packs against residues lining the active site pocket distal to the catalytic center. In FluA wild-type, BXA makes van der Waals contacts with Ala20 and Tyr24 from the C-terminal end of helix α2 and Lys34, Ala37 and Ile38 from helix α3 (Fig. 4A). Tyr24 binds across the two wings of the compound, contacting both the oxazino on the head group and the difluorobenzene on the tail-group (Fig. 4A). These interactions together with a salt-bridge between Glu26 and Lys34 stabilises the α2-α3 loop that is otherwise particularly mobile^21^. In FluB wild-type, very similar interactions are made but with slightly different residues Thr20, Phe24, Met34, Asn37 and Ile38 (Fig. 5A). The reduced size of Phe24 compared with Tyr24 allows Arg85 to change conformation and contact the compound in FluB but not FluA (Fig. 5). Conserved Ile38 intimately packs into the V of the tail-group in identical fashion in both FluA and FluB, making van der Waals contacts via its CG1 and CD1 atoms. When this residue is mutated to the more polar threonine, the structure is essentially unchanged in both the FluA and FluB structures except for the side-chain of the residue (Figs 4–6). The side-chain of T38 is orientated such that its methyl-group is in exactly the same position as the CG1 of Ile38, whereas the OH group is orientated towards helix α3 allowing it to make two hydrogen bonds to the main-chain carbonyl oxygens of Met34 and Leu35 (Figs 6, 7). Thus, substitution of Ile38 by Thr38 reduces slightly the van der Waals interaction with the compound since threonine lacks the CD1 methyl group of isoleucine. However, another difference is revealed if we compare the structures of the unbound and bound forms of the wild-type and mutant endonuclease (Fig. 7). For the wild-type FluB endonuclease, the I38 side-chain conformation does not change upon ligand binding (Fig. 7A). A similar observation is true for FluA endonuclease (not shown). For the FluB I38T mutant in the absence of ligand, the threonine side-chain changes rotamer (Fig. 7B). The methyl-group is now in the position of the CG2 of the isoleucine and the hydroxyl group is only able to make one hydrogen bond with the carbonyl oxygen of Met34. Furthermore, in the unbound form, Met34 is orientated into the pocket and also has to change rotamer to accommodate the ligand in which case it packs against the heterocyclic fluorines of the tail-group (Fig. 7B). Thus binding of BXA to the T38 variant endonuclease requires local induced fit rearrangements, whereas binding to I38 does not.

## Discussion

In this study, we characterise amino acid substitutions detected in the baseline and treatment-emergent variant monitoring of two different clinical trials in which influenza infected patients received the novel endonuclease inhibitor BXM. We demonstrate that mutations of I38 in PA are the major pathway for reduced susceptibility to BXM on these variants only occurred in patients treated with BXM. The I38T had the largest effect in A/H1N1 and A/H3N2 viruses, which emerged in two (1.1%) of 182 in the phase 2 and in 15 (19.5%) of 77 of treated patients in the pediatric study, respectively, increasing the EC_50_ by approximately 30- to 50-fold, with lesser effects for I38F and I38M. Polymorphic I38V did not affect BXA sensitivity. The impact of I38 substitutions was less pronounced in FluB than in FluA. We also identified E23K, A37T and polymorphic A36V, as PA mutations that have a significant but lesser impact on compound susceptibility. All cited mutations arise from single nucleotide changes in the viral genome. Consistent with these observations, our co-crystal structures of FluA and FluB endonucleases with BXA show that all these residues line the active site pocket where BXA binds and mutations of them could influence BXA affinity. In particular, our structural studies comparing the wild-type and I38T mutant show that BXA binds in an almost identical manner with differences restricted locally to the I/T side-chain change only. I38 packs intimately into the V shape of the BXA tail-group whereas T38 makes slightly less contact due to the lack of the extra methyl-group. Furthermore, unlike I38, which does not change conformation upon BXA binding, T38 (and M34 in the case of FluB) have to change rotamer to accommodate the inhibitor binding. These two effects (less favourable van der Waals packing and induced fit change of threonine rotamer) plausibly account for the reduced affinity of the inhibitor for the I38T variant and hence the lower susceptibility of viruses bearing this mutation. This reduced affinity is reflected in the ten degree lower melting temperature of the BXA-bound I38T endonuclease compared to the wild-type, indicative of a significantly less stable enzyme-inhibitor complex. We also found that E199G in FluA, located at the beginning of the linker between the PA N- (endonuclease) and C-terminal domains, slightly reduced BXA susceptibility by 4.46-fold. This residue is in the linker between N- and C-terminal regions of PA and is not in the construct crystallised which stops at E198. In the FluA crystal structures E198 makes a salt bridge with Arg124 from a neighbouring molecule so no clear conclusions can be made about the possible role of E199 residue in BXA binding in the context of the complete polymerase.

Although I38T mutation confers reduced sensitivity to BXA, we also show that recombinant influenza A viruses bearing this mutation have a significantly reduced fitness in canine and human cells, as measured by growth curves. Given that the I38 in PA protein is >99.9% conserved in sequenced strains (Supp. Table 2) and is positioned to likely interact with the RNA substrate, perhaps by base stacking, we speculate that the substitution I38T to a less hydrophobic residue reduces the affinity to RNA as well as to BXA. This is consistent with our observation that the *in vitro* activity of the isolated endonuclease domain of both influenzas A and B is reduced for the I38T variant compared to wild-type. The reduction of susceptibility to BXM for the I38T mutant is less for FluB compared to FluA. Since the direct effect of the substitution, as observed in the crystal structures, is essentially the same in FluA and FluB this suggests that the contribution of I38 to the affinity of the compound is proportionally less in FluB than in FluA. This could perhaps be explained by the correspondingly greater role of Met34 in interacting with the compound in FluB compared to Lys34 in FluA. Interestingly no FluB variants were detected that mutated Met34, which is presumably of critical importance to endonuclease function. That Met34 is also important for substrate RNA binding could explain why, in the case of FluB, the I38T mutation has little impact on viral fitness. In contrast, for FluB, the I38F mutation does significantly impact viral fitness possibly because the larger phenylalanine side-chain more severely impacts productive substrate RNA binding.

In future studies, exploration of compensatory substitutions that recover the replicative capacity of I38T viruses will be required. This is in the light of the previous worldwide prevalence of NA inhibitor-resistant viruses^35^ and reports on compensatory substitutions to enhance the reduced fitness of NA inhibitor-resistant viruses^36,37^. However, compensatory substitutions have not yet been observed with variants such as A20S + I38F or I38T + E623K (Supp. Fig. 1).

In summary, this report shows that a minority of treated patients select for influenza variants that escape the high potency of BXA and we give a structure-based mechanistic explanation of how the most prominent of these, I38T, reduces susceptibility to the drug. However, these variant viruses have reduced fitness and may not propagate in the absence of the selective pressure imposed by the compound. It remains to be seen how this might impact future use of the drug. Importantly, no correlation of the emergence of I38T or other substitutions with clinical influenza symptoms or relapse/persistence of fever has emerged so far. However, further large scale clinical investigations will be needed to shed more light on the relationship between the treatment-emergent variants and clinical symptoms. Finally, our results provide markers of reduced susceptibility to BXM which will be of value in future surveillance of treated populations.

## Methods

### Compounds

Baloxavir acid (S-033447; BXA) was synthesized and supplied by Shionogi & Co., Ltd, and favipiravir was purchased from PharmaBlock Sciences, Inc. (Nanjing, China). BXA and favipiravir were dissolved in dimethyl sulfoxide (Nacalai Tesque, Inc.) to prepare 10 mM and 100 mM solutions, respectively. Oseltamivir acid was purchased from Toronto Research Chemicals Inc. (Toronto, Ontario, Canada) and dissolved in distilled water (DW) to prepare 10 mM solution.

### Cells and viruses

Canine kidney MDCK cells were obtained from European Collection of Cell Cultures. Human quasi-diploid tumor RPMI2650 and human embryonic kidney 293 T cells were provided by American Type Culture Collection. MDCK and RPMI2650 cells were maintained in minimal essential medium (MEM) (Thermo Fisher Scientific, Inc.) supplemented with 10% fetal bovine serum (FBS) (Sigma-Aldrich Co., Ltd.) and 100 µg/mL kanamycin (Thermo Fisher Scientific, Inc.). 293 T cells were cultured in Dulbecco’s modified Eagle’s medium with 10% FBS and 100 µg/mL kanamycin. Eight plasmids-based reverse genetics technique was employed to generate recombinant viruses as described^38^. The plasmid set of rgA/WSN/33 (H1N1) and empty vector pHW2000 were provided by Dr. Robert Webster at St. Jude Children’s Research Hospital. The plasmids for the generation of rgA/Victoria/3/75 and rgB/Maryland viruses were constructed with the pHW2000 by standard molecular biology techniques. The primer sequences used are available upon request. Co-culture of MDCK and 293 T cells were transfected with the eight plasmids and incubated 48 to 72 hours, followed by propagation of the viruses in MDCK cells. The PA sequences of the recombinant viruses were verified by Sanger sequencing. Viral titers were determined by standard tissue culture infectious dose (TCID)_50_ assay or plaque-forming unit (PFU) assay in MDCK cells.

### Plaque reduction assay

MDCK cells seeded in 12-well plates were infected with approximately 50 PFU/well of the viruses, followed by incubation at 33 °C in 5% CO_2_ incubator for 1 hour. The infected cells were washed twice with MEM and overlaid with MEM containing the BXA or favipiravir, 0.8% Difco Noble Agar (Becton, Dickinson and Company) and 3 μg/mL trypsin (Sigma-Aldrich Co., Ltd.). After incubation at 33 °C in 5% CO_2_ incubator for 3 days, the cells were fixed with 0.5 mL of 5:1 (v/v) mixture of methanol: acetic acid at room temperature for 30 minutes. The cells were stained with 300 μL of 0.5% amido black solution (Wako Pure Chemical Industries, Ltd) and incubated at room temperature for 30 minutes, followed by counting plaque numbers and calculating the concentration achieving 50% inhibition of plaque formation (EC_50_) by using software XLfit version 5.3.1.3 (ID Business Solutions). FC was calculated by dividing EC_50_ of each tested virus to EC_50_ of the cognate wild-type virus.

### Neuraminidase inhibition assay

Oseltamivir acid was serially diluted in MES assay buffer [32.5 mmol/L MES and 4 mmol/L CaCl_2_ in DW (pH 6.5 adjusted with 4 N NaOH)]. To prepare NA enzyme solution, virus stocks were inactivated by 0.1% NP-40, and diluted with MES assay buffer. Ten μL of the oseltamivir acid solution and 10 μL of the NA enzyme solution were mixed and incubated at 37 °C for 30 minutes, followed by addition of 30 μL of 100 μmol/L 2′-(4-Methylumbelliferyl)-α-D-N-acetylneuraminic acid sodium salt hydrate (MUNANA; Sigma-Aldrich Co., Ltd.). The reaction mixtures were incubated at 37 °C for 60 minutes, and the reaction was stopped by addition of 150 μL of stop solution [0.1 mol/L glycine and 25% ethanol (pH 10.7 adjusted with 4 N NaOH)]. The fluorescence intensity was measured with a microplate reader EnVision 2103 (PerkinElmer Inc.) at excitation wavelength of 355 nm and an emission wavelength of 460 nm, followed by calculation of IC_50_ values with XLfit software. FC was calculated by dividing IC_50_ of each tested virus to IC_50_ of the cognate wild-type virus.

### Evaluation of replicative capacity of the viruses

MDCK or RPMI2650 cells were seeded on 24-well plates 1 day prior to infection. MDCK cells were infected with 10 TCID_50_/well of the recombinant viruses. For the infection of RPMI2650 cells, 10 TCID_50_/well of rgA/WSN/33 (H1N1) and 100 TCID_50_/well of the rgA/Victoria/3/75 (H3N2) or rgB/Maryland/1/59 viruses were used. The infected cells were incubated at 37 °C in 5% CO_2_ incubator for 1 hour, and then washed with MEM, followed by addition of MEM containing 3 μg/mL trypsin and incubation at 37 °C in the 5% CO_2_ incubator. The culture supernatants of MDCK or RPMI2650 cells were collected at the indicated time points, and viral titers (logTCID_50_/mL) were determined on MDCK cells.

### Expression and purification of PA endonuclease domains

The DNA coding for PA N-terminus 1–198 from A/California/04/2009/H1N1 (FluA) and 1–197 from B/Memphis/13/03 (FluB) was subcloned in the expression vector pETM11 (Gunter Stier, EMBL). Point mutations encoding for the substitution I38T in both FluA and FluB were introduced by site-directed mutagenesis. Wild-type and mutant endonuclease were expressed and purified as described^21,32^. Protein was stored in 20 mM Tris/HCl pH 7.5, 100 mM NaCl, 5 mM 2-mercaptoethanol supplemented with 2 mM MnCl_2_ and 2 mM MgCl_2_ unless otherwise stated.

### Thermal stability assay

The effect of bound BXA on the thermal stability of the wild-type and mutant FluA and FluB endonucleases was determined as described^14,39^. Briefly, assays were performed with 13 µM PA-Nter in 20 mM Tris/HCl pH 7.5, 100 mM NaCl, in the presence or absence of 0–1.3 mM of BXA and SYPRO Orange dye (Invitrogen). The dye was excited at 490 nm and the emission light was recorded at 575 nm while the temperature was increased by 1 °C per minute in the range 25–95 °C. The recorded fluorescence was plotted against the temperature using GraphPad Prism (GraphPad Software, USA). The inflection point of the denaturation curves represented the Tm values.

### Endonuclease assay

RNA cleavage was performed by incubating purified PA proteins with 1 μM FAM-labelled 42 nt ssRNA substrate (FAM-Ex-5-UAUACUGACUACAAUCCCCACUCCUAUACCUCUGCUUCUGCU, IBA Lifesciences) for 2 h at 37 °C in a final volume of 10 µL. FluA WT was used at either 0.5 or 5 µM, and FluB at 5 or 50 µM, whereas their respective mutant forms were used at 10-fold higher concentrations. For inhibition assays, protein samples were pre-incubated for 30′ at room temperature with increasing concentrations on BAX (inhibitor:protein molar ratios of 0.1:1, 0.2:1, 0.5:1 and 1:1) prior activity assay. The reaction buffer was 50 mM Tris-HCl pH 8.0, 100 mM NaCl, 10 mM β-mercaptoethanol, 2.5 mM MnCl_2_. Reactions were stopped by addition of loading dye with EDTA at a final concentration of 1 mM and boiling of samples at 98 °C for 3′. The reaction products were loaded on 8 M urea polyacrylamide gels (20%). FAM fluorescence was detected at 520 nm using a UV-transilluminator as excitation source and standard emission filter in Gel Doc™ XR+ (BioRad). Gels were exposed using automatic settings for intense bands, and each panel was acquired at a single exposure time: 14.2 sec for top left, 10.7 sec for top right, 10.6 sec for bottom left and 10.2 sec for bottom right. Image processing was carried out using Image Lab software, version 6.0.0.25 (BioRad): the original image display (white for bands signal, black for background) was inverted and the gels cropped by removing the gel wells and band-less edges only, prior to exporting at 600 dpi for publication.

### Crystallization, data collection, and refinement

FluA or FluB endonuclease co-crystals with BXA were prepared at 20 °C using either the hanging-drop (FluA WT) or sitting-drop technique (FluA I38T, FluB). Each protein, at 15–17 mg/ml, was incubated with 10-fold molar excess of BXA for 30 min at RT, mixtures were centrifuged at RT for 5 minutes at 12000 g, and soluble fraction was used for crystallization trials (final protein concentration 8–10 mg/ml). Crystals grew over a period of 2–4 weeks in the following conditions:

FluA WT BXA: 0.3 M (NH4)_2_SO4, 0.1 M ADA pH 6.5, 28% (w/v) PEG 8000;

FluA I38T BXA: 0.2 M (NH4)_2_SO4, 0.1 M Na(CH3)_2_AsO_2_ pH 6.5, 30% (w/v) PEG 8000;

FluB WT BXA: 0.2 M NaCl, 0.1 M Na(CH3)_2_AsO_2_ pH 6.5, 2 M (NH4)_2_SO4;

FluB I38T BXA: 0.2 M CaCl_2_, 0.1 M MES pH 6.0, 20% (w/v) PEG 6000.

FluB I38T-no ligand: 0.04 M KH_2_PO4, 16% (w/v) PEG 8000, 20% (v/v) glycerol

Single crystals were fished and washed in a reservoir solution supplemented with 20% glycerol for cryoprotection (except FluB I38T-no ligand) and immediately frozen in liquid nitrogen. X-ray diffraction data were collected at a wavelength of 0.966 Å on beamline ID30A-1, 0.968 Å on beamline ID30A-3 of 1.254 Å on ID29 at the European Synchrotron Radiation Facility (ESRF, Grenoble, France). Data processing were carried out using XDS^40^ and scaling with XSCALE^40^ or AIMLESS^41^. The structures were solved by molecular replacement with PHASER^42^ using as search models PDB:4AWK for FluA or PDB:5FML for FluB. Structure refinement was performed using cycles of phenix.refine^43^ or REFMAC5^44^ and manual building in Coot^45^. MolProbity^46^ and the wwwPDB validation tools^47^ were used for structure validation. Final data collection and refinement statistics are shown in Table 2. Structure figures were prepared using PyMOL (The PyMOL Molecular Graphics System; Schrödinger LLC; www.pymol.org).

### Ethics statement

The clinical trials were registered in Japan Pharmaceutical Information Center Clinical Trials Information with reference numbers Japic CTI-153090 and Japic CTI-163417 (http://www.clinicaltrials.jp/user/cteSearch_e.jsp). Written informed consent was obtained from all the patients in clinical trials, and all methods related to clinical samples were derived according to standard operating procedures in accordance with the protocol approved by the institutional review board (IRB), all applicable regulatory requirements, and the current Good Clinical Practice (GCP) guidelines.

### Data availability

Coordinates and structure factors for PA-FluA WT and I38T and PA-FluB WT and I38T in complex with BXA have been deposited in the wwPDB under accession codes 6FS6, 6FS7, 6FS8 and 6FS9 respectively and 6FSB for the ligand-free PA-FluB I38T variant.

## Electronic supplementary material

Supplementary Information
