# Supplementary material for: Characterization of influenza virus variants induced by treatment with the endonuclease inhibitor baloxavir marboxil
Source: Sci Rep. 2018 Jun 25;8:9633. doi: 10.1038/s41598-018-27890-4 (PMC6018108; doi:10.1038/s41598-018-27890-4)
Supplement: Supplementary file 1 — Supplementary Information [file 41598_2018_27890_MOESM1_ESM.pdf]

Characterization of influenza virus variants induced by treatment with the endonuclease inhibitor Baloxavir Marboxil

Shinya Omoto[a#*], Valentina Speranzini[b#], Takashi Hashimoto[a], Takeshi Noshi[a], Hiroto Yamaguchi[a], Makoto Kawai[a], Keiko Kawaguchi[a], Takeki Uehara[a], Takao Shishido[a], Akira Naito[a], and Stephen Cusack[b*]

[a]Shionogi & Co., Ltd., Osaka, Japan

[b]European Molecular Biology Laboratory, Grenoble Outstation, Grenoble, France

[#] Equal contribution to this work

* To whom correspondence should be addressed:

Dr. Stephen Cusack, EMBL Grenoble Outstation, 71 Avenue des Martyrs, CS 90181, 38042 Grenoble Cedex 9, France

Tel: (33) 4 76 20 7238, Fax: (33) 4 76 20 7199, E-mail: cusack@embl.fr

Dr. Shinya Omoto, Shionogi Pharmaceutical Research Center, 3-1-1, Futaba-cho, Toyonaka, Osaka 561-0825, Japan

Tel: +81-6-6331-7266, Fax : +81-6-6332-6385, E-mail: shinya.oomoto@shionogi.co.jp

Supplementary Tables and Figures

Position	28			36			63			356		
	AA	N	Freq	AA	N	Freq	AA	N	Freq	AA	N	Freq
A/H1N1	P	4,975	76.43	A	6,506	99.97	V	6,476	99.51	R	6,472	99.17
	L	1,529	23.49	V	2	0.03	I	30	0.46	K	54	0.83
	S	4	0.06				A	1	0.02			
	Q	1	0.02				F	1	0.02			
	Total	6,509	100	Total	6,508	100	Total	6,508	100	Total	6,526	100
A/H3N2	L	5,223	99.33	A	5,263	100	V	5,213	98.94	R	5,232	99.32
	S	23	0.44				I	56	1.06	K	36	0.68
	P	6	0.11									
	M	5	0.1									
	Q	1	0.02									
	Total	5,258	100	Total	5,263	100	Total	5,269	100	Total	5,268	100
B	P	1,939	100	F	1,943	100	A	1,945	99.95	L	1,964	100
							V	1	0.05			
	Total	1,939	100	Total	1,943	100	Total	1,946	100	Total	1,964	100

AA = amino acid; N = numbers of sequence samples; Freq = frequency (%). Sequence data as of August 8 in 2014 were collected from NCBI influenza virus resources and analyzed frequency of AA variants at the indicated position.

Supplementary Table 1. Frequency of amino acid variants at the position 28, 36, 63 and 356 in PA proteins which were detected in the baseline variant monitoring in a phase 2 and pediatric study from H1N1, H3N2 and type B viruses-infected subjects.

Position	23			37			38			199		
	**AA**	**N**	**Freq**	**AA**	**N**	**Freq**	**AA**	**N**	**Freq**	**AA**	**N**	**Freq**
A/H1N1	E	6,505	99.95	A	6,505	99.95	I	6,502	99.92	E	6,497	99.92
	N	2	0.03	S	2	0.03	V	4	0.06	D	4	0.06
	G	1	0.02	T	1	0.02	M	1	0.02	K	1	0.02
	Total	6,508	100	Total	6,508	100	Total	6,507	100	Total	6,502	100
A/H3N2	E	5,238	100	A	5,264	100	I	5,261	99.96	E	5,284	99.98
							M	2	0.04	K	1	0.02
	Total	5,238	100	Total	5,264	100	Total	5,263	100	Total	5,285	100
B	E	1,938	100	N	1,943	100	I	1,943	100	G	1,968	100
	Total	1,938	100	Total	1,943	100	Total	1,943	100	Total	1,968	100

AA = amino acid; N = numbers of sequence samples; Freq = frequency (%). Sequence data as of August 8 in 2014 were collected from NCBI influenza virus resources and analyzed frequency of AA variants at the indicated position.

Supplementary Table 2. Frequency of amino acid variants at the position 23, 37, 38 and 199 in PA proteins which were detected in the treatment-emergent variant monitoring in a phase 2 and pediatric study from H1N1, H3N2 and type B viruses-infected subjects.

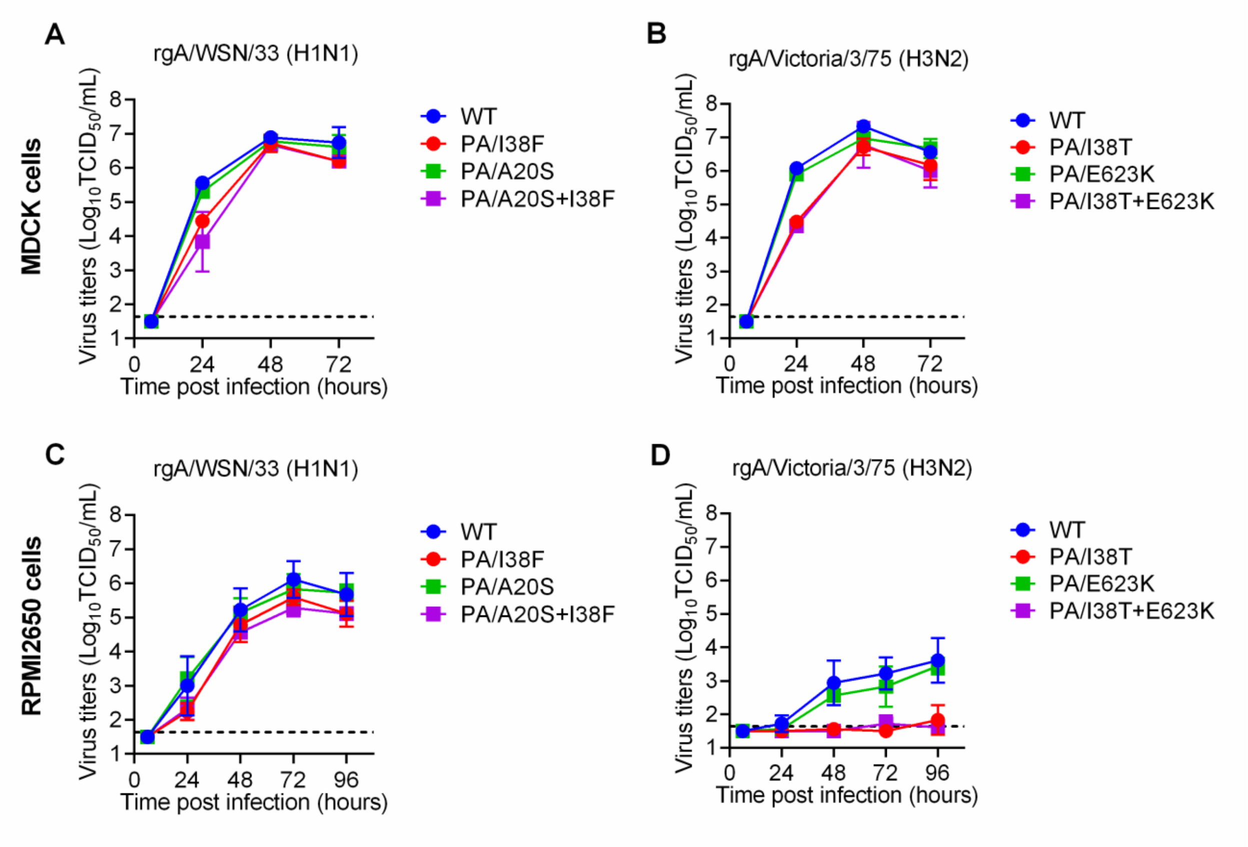

Supplementary Figure 1. Replicative capacity of variant viruses with indicated AA substitutions in PA protein. Canine MDCK cells (A and B) or human RPMI2650 cells (C to D) were infected with WT or mutant viruses based on rgA/WSN/33 (H1N1) (A and C) or rgA/Victoria/3/75 (H3N2) (B and D). The culture supernatants were collected at indicated time points and virus titers ($TCID_{50}$/mL) were determined in MDCK cells. Each symbol represents the mean and standard deviation of triplicate experiments. The lower limit of quantification of the virus titers was indicated by a dashed line.

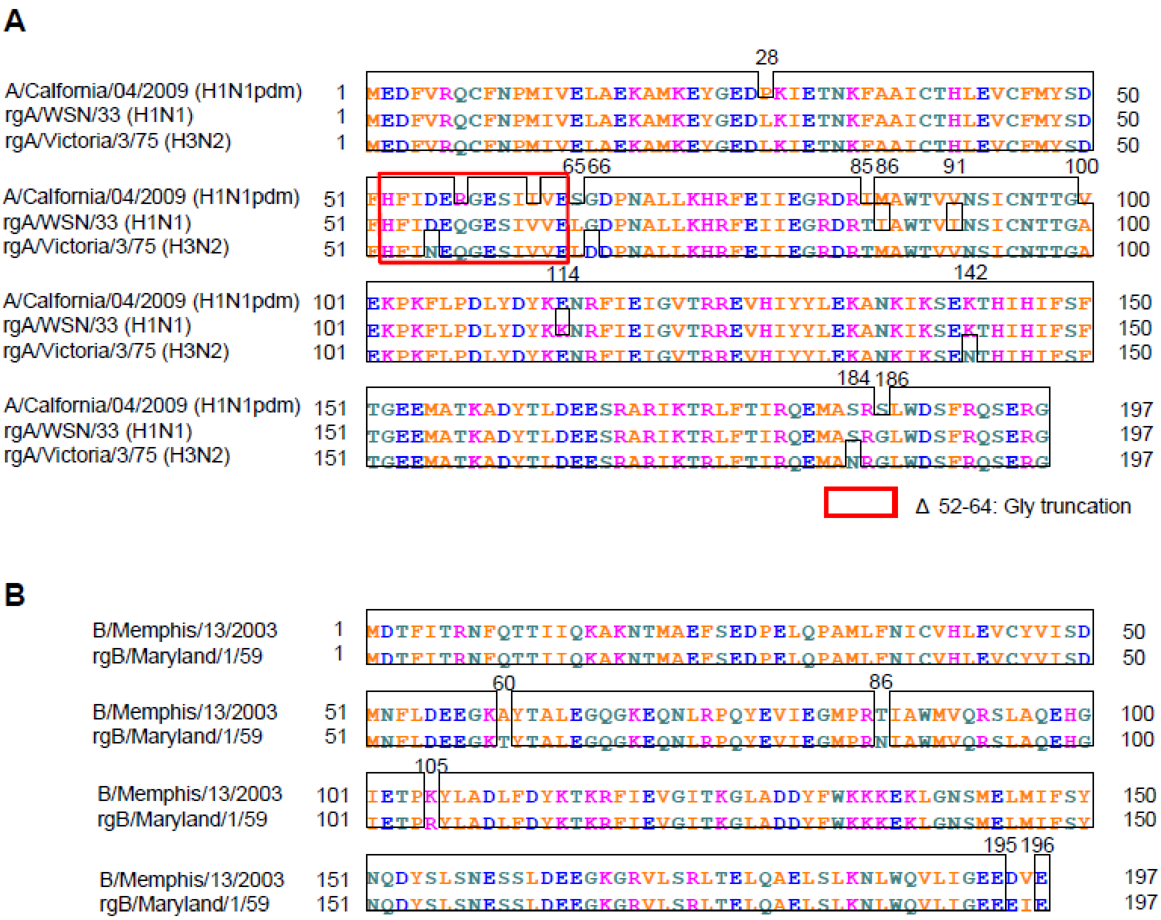

Supplementary Figure 2. Amino acid sequence alignment of PA N-terminal cap-dependent endonuclease domain (A) Sequence alignment of A/California/04/2009 (H1N1pdm), rgA/WSN/33 (H1N1) and rgA/Victoria/3/75 (H3N2) from top to bottom. The red outline indicates the truncated region (52 to 63 aa) in the crystallised construct. (B) Sequence alignment of B/Memphis/13/2003 (upper) and rgB/Maryland/1/59 (lower). In (A) and (B), sequences are identical except where indicated by residue number. Alignment was generated by Genetyx-Win, version 11 (Genetyx Corporation, Tokyo, Japan).

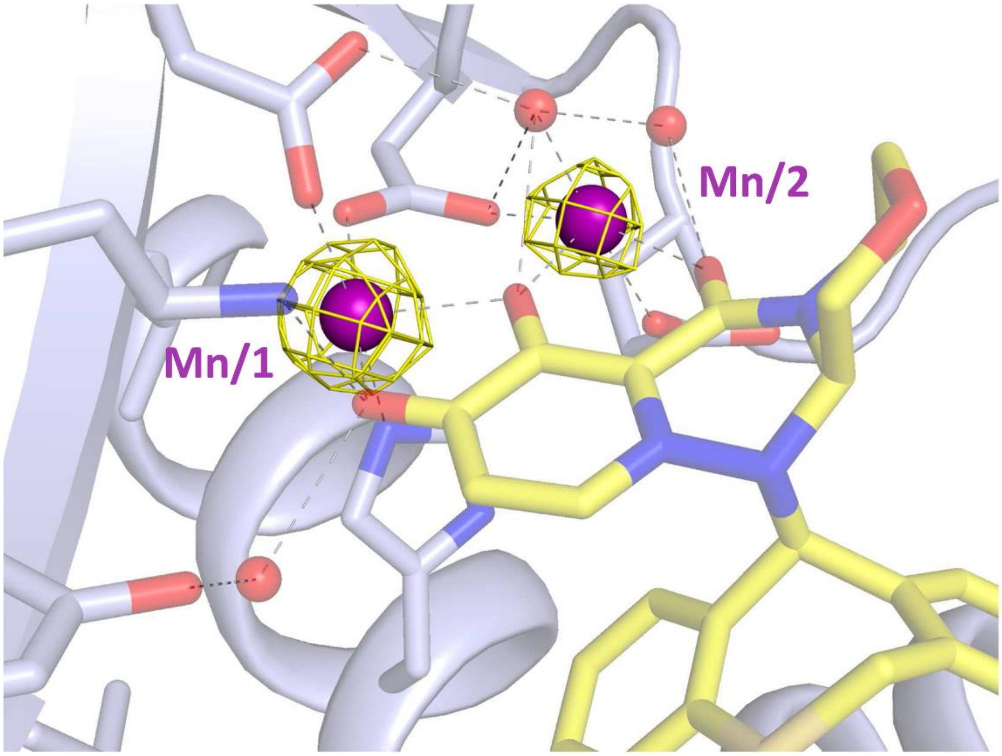

Supplementary Figure 3. BXA interacts with PA active site by chelating two metal ions

Structural detail of BXA (yellow sticks) interacting with manganese ions at PA active site. The anomalous scattering map (yellow mesh, 4.0 σ) of the metal ions allowed the identification of manganese in both sites. The lower signal at site 2 suggests that a fraction of the protein molecules may bind magnesium instead of manganese, as PA was purified in presence of both ion species. In the FluA I38T structure, anomalous peak heights vary from 14.8-19.2 σ for site 1 and from 8.4-10.6 for site 2.
